# Association of Prenatal, Early Postnatal, or Current Exposure to Secondhand Smoke With Attention-Deficit/Hyperactivity Disorder Symptoms in Children

**DOI:** 10.1001/jamanetworkopen.2021.10931

**Published:** 2021-05-20

**Authors:** Li-Zi Lin, Shu-Li Xu, Qi-Zhen Wu, Yang Zhou, Hui-Min Ma, Duo-Hong Chen, Gong-Bo Chen, Hong-Yao Yu, Bo-Yi Yang, Xiao-Wen Zeng, Li-Wen Hu, Guang-Hui Dong

**Affiliations:** 1 Guangdong Provincial Engineering Technology Research Center of Environmental Pollution and Health Risk Assessment, Department of Occupational and Environmental Health, School of Public Health, Sun Yat-sen University, Guangzhou, China; 2 State Environmental Protection Key Laboratory of Environmental Pollution Health Risk Assessment, South China Institute of Environmental Sciences, Ministry of Environmental Protection, Guangzhou, China; 3 State Key Laboratory of Organic Geochemistry and Guangdong Key Laboratory of Environmental Protection and Resources Utilization, Guangzhou Institute of Geochemistry, Chinese Academy of Sciences, Guangzhou, China; 4 Department of Air Quality Forecasting and Early Warning, Guangdong Environmental Monitoring Center, State Environmental Protection Key Laboratory of Regional Air Quality Monitoring, Guangdong Environmental Protection Key Laboratory of Atmospheric Secondary Pollution, Guangzhou, China

## Abstract

**Importance:**

Few studies have investigated the association between the exposure window (prenatal, early postnatal, and current period) of secondhand smoke (SHS) and attention-deficit/hyperactivity disorder (ADHD) symptoms and subtypes in children.

**Objective:**

To evaluate the associations of prenatal, early postnatal, or current SHS exposure with ADHD symptoms and subtypes among school-aged children.

**Design, Setting, and Participants:**

In this cross-sectional study, 48 612 children aged 6 to 18 years from elementary and middle schools in Liaoning province, China, between April 2012 and January 2013 were eligible for participation. Data on SHS exposure and ADHD symptoms and subtypes for each child were collected via questionnaires administered to parents or guardians by school teachers. Data were analyzed from September 14 to December 2, 2020.

**Main Outcomes and Measures:**

The ADHD symptoms and subtypes (inattention, hyperactivity-impulsivity, and combined) were measured based on a validated tool developed from the *Diagnostic and Statistical Manual of Mental Disorders* (Fourth Edition). Generalized linear mixed models were evaluated to estimate the association of SHS exposure with ADHD symptoms and subtypes.

**Results:**

A total of 45 562 participants completed the questionnaires and were included in this study (22 905 girls [50.3%]; mean [SD] age, 11.0 [2.6] years; 2170 [4.8%] with ADHD symptoms). Compared with their unexposed counterparts, children who were ever exposed (odds ratio [OR], 1.50; 95% CI, 1.36-1.66) or always exposed to SHS (OR, 2.88; 95% CI, 2.55-3.25) from pregnancy to childhood had higher odds of having ADHD symptoms and subtypes (ORs ranged from 1.46 [95% CI, 1.31-1.62] to 2.94 [95% CI, 2.09-4.13]). Compared with their unexposed counterparts, children with SHS exposure had higher odds of having ADHD symptoms when exposed in the prenatal period (OR, 2.28; 95% CI, 2.07-2.51), early postnatal period (OR, 1.47; 95% CI, 1.29-1.68), or current period (OR, 1.20; 95% CI, 1.09-1.31). Compared with their unexposed counterparts, children whose fathers smoked 10 or more cigarettes/d on both weekdays and weekends had higher odds of having ADHD symptoms and subtypes (ORs ranged from 1.48 [95% CI, 1.28-1.70] to 2.25 [95% CI, 1.29-3.93]).

**Conclusions and Relevance:**

Being exposed to SHS from pregnancy to childhood was associated with higher odds of having ADHD symptoms and subtypes among school-aged children, and the associations were somewhat stronger for SHS exposure during prenatal and early postnatal periods. Our findings highlight the important public health implications of reducing SHS exposure, which may decrease the health and economic burdens of individuals with ADHD.

## Key Points

**Question:**

Are there associations between prenatal, early postnatal, or current exposure to secondhand smoke (SHS) and symptoms or subtypes of attention-deficit/hyperactivity disorder (ADHD) among children?

**Findings:**

In this cross-sectional study of 45 562 school-aged children in China, SHS exposure from pregnancy to childhood was associated with higher odds of having ADHD symptoms and subtypes. When considering the timing of SHS exposure, the associations were somewhat stronger in the prenatal and early postnatal periods.

**Meaning:**

These findings support the association of SHS exposure with ADHD-related symptoms among children and highlight the importance of strengthening public health efforts to reduce SHS exposure.

## Teaser

This cross-sectional study analyzes data collected via questionnaires to assess whether associations exist between prenatal, early postnatal, or current exposure to secondhand smoke and symptoms or subtypes of attention-deficit/hyperactivity disorder among school-aged children in China.

## Introduction

Attention-deficit/hyperactivity disorder (ADHD), characterized by a persistent pattern of inattention, hyperactivity-impulsivity, or both, is a prevalent, impairing condition that creates a substantial burden for both individuals and society.^1^ One recent umbrella review suggested that maternal smoking during pregnancy was strongly associated with the development of ADHD symptoms in their children.^2^ When mothers inhale smoke from cigarettes, nicotine distilled from the tobacco is rapidly metabolized in the liver to cotinine, which may disrupt the maturation of the central nervous system, resulting in later development of ADHD symptoms in their offspring.^3^ In China, however, the prevalence of smoking among women is low, whereas among men, it is high (2.1% vs 50.5% in 2018).^4^ Approximately 40% of Chinese women have been exposed to secondhand smoke (SHS), primarily in the home environment.^4^ Therefore, SHS exposure may be an important environmental factor associated with ADHD in China.

Although most studies addressing SHS and ADHD symptoms have evaluated prenatal exposure (eTable 1 in the Supplement),^5,6,7,8^ postnatal SHS exposure may also induce ADHD deficits because the human brain continues to develop during the postnatal period.^9^ Among studies considering both prenatal and postnatal SHS periods, 3 cohort studies and 1 case-control study suggested that SHS exposure from pregnancy to childhood was associated with ADHD symptoms.^6,10,11,12^ However, those studies were unable to distinguish between the postnatal exposure during early childhood and later childhood. Therefore, the association of the specific timing of SHS exposure with ADHD symptoms has not yet been clarified. Although paternal smoking is the main source of SHS,^4^ few fathers report prenatal smoking abstinence and continue to smoke during pregnancy, after childbirth, or both.^4,13,14^ Detailed investigations of paternal smoking are still warranted.

Children with different ADHD subtypes experience different symptoms, with subtype-specific effects associated with developing distinct cognitive characteristics, trajectories of symptom persistence, and patterns of comorbidity.^15,16,17^ Therefore, it is also important to study the associations between various windows of SHS exposure and subtypes of ADHD symptoms, although different SHS exposure windows have been seldom studied.

We conducted the present study to examine the associations of SHS exposure with ADHD symptoms and subtypes in school-aged children by considering the timing of the exposure. We hypothesized that the associations between SHS exposure and ADHD symptoms may differ with different exposure timing and among the various subtypes of ADHD symptoms.

## Methods

### Study Population and Overall Design

We used the cross-sectional data embedded in the second wave of the Seven Northeastern Cities study, which was conducted between April 2012 and January 2013. For our sampling strategy, we generated a representative sample by randomly selecting half of the 14 cities in Liaoning province, located in northeastern China. We included 24 urban districts from the 7 selected cities, randomly choosing 1 elementary school and 1 middle school. From each grade level of the included schools, we invited students from 1 or 2 classrooms who lived within the study district for at least 2 years before the start of this study to participate in our survey. We followed the Strengthening the Reporting of Observational Studies in Epidemiology (STROBE) reporting guideline for cross-sectional studies. This study was approved by the Ethical Review Committee for Biomedical Research, Sun Yat-sen University. All children and their parents provided written informed consent that was obtained in a manner consistent with the Common Rule requirements. No one received compensation or was offered any incentive for participating in this study.

### Questionnaires

We obtained individuals’ information via questionnaires. We used active communication techniques to incentivize permission from parents with the help of the teachers and principals of the included schools. We organized face-to-face meetings between our research staff (including L.-W.H. and G.-H.D.) and the teachers and principals to enable them to understand the study aims, proposed methods, and study procedures in detail. We provided structural procedures for the teachers who were required to distribute and collect the questionnaires and envelopes and forms to record the questionnaire responses. Teachers were responsible for explaining the study aim to parents, obtaining informed consent, and collecting the questionnaires during regular parent-teacher conferences. Parents were allowed to fill out the questionnaires during the conference or to take them home and return them in a sealed envelope. Parents were also given the opportunity to decline to consent and to refuse to join the study.

### ADHD Symptom Evaluation

We asked parents to fill out both the symptom inventory scale of ADHD (SIS-ADHD) and the Conners Abbreviated Symptom Questionnaire (C-ASQ) to measure ADHD symptoms in all children. The Chinese version of the SIS-ADHD^18^ was developed based on the *Diagnostic and Statistical Manual of Mental Disorders* (Fourth Edition) (*DSM-IV*).^19^ The scale consists of 18 queries categorized as 9 symptoms of inattention and 9 symptoms of hyperactivity-impulsivity. Each item or symptom was rated on a 4-point Likert scale (0, never or rare; 1, sometimes; 2, often; and 3, very often). Children with ADHD symptoms were defined as presenting often or very often with 6 or more symptoms of inattention or with 6 or more symptoms of hyperactivity-impulsivity. The ADHD symptoms were further classified into 3 subtypes: (1) inattentive (ADHD-I), defined as presenting often or very often with 6 or more symptoms of inattention; (2) hyperactivity-impulsivity (ADHD-HI), defined as presenting often or very often with 6 or more symptoms of hyperactivity-impulsivity; and (3) combined (ADHD-C), defined as presenting often or very often with 6 or more symptoms of inattention and with 6 or more symptoms of hyperactivity-impulsivity. The sensitivity and specificity of this psychometric property were 91% and 97%, respectively.

We reevaluated ADHD symptoms by using the Conners Hyperkinesis Index based on the validated Chinese version of the C-ASQ.^20^ The C-ASQ is a set of 10 checklist items rated on a 4-point Likert scale (0, never or rare; 1, sometimes; 2, often; and 3, very often). The Conners Hyperkinesis Index is a sum of the total score and ranges from 0 to 30, with a score higher than 15 defined as having ADHD symptoms. The sensitivity and specificity were 76.0% and 92.2%, respectively.

### Assessment of SHS Exposure

We collected information on SHS exposure for different exposure windows, including the prenatal, postnatal (ie, first 2 years of life), and current periods. We defined having prenatal SHS exposure based on an affirmative answer to the question, “Did anyone who lived with the mother during her pregnancy smoke anywhere inside the house?” We defined having postnatal SHS exposure based on an affirmative answer to the question, “Did anyone who lived with the child during his or her first 2 years smoke anywhere inside the house?” We collected information on the current number of cigarettes smoked inside the house per day during weekdays and weekends by all family members who lived with the child. We defined having current SHS exposure if any family member who lived with the child smoked cigarettes. Therefore, the SHS exposure for the different exposure windows was a binary variable encoded as 0 for no and 1 for yes. We further grouped SHS exposure from pregnancy to childhood into 3 categories: unexposed, ever exposed, and always exposed. We analyzed current paternal smoking on weekdays or weekends because this was the main source of children’s SHS exposure, and we grouped the number of cigarettes per day into 4 categories (0, 1 to <5, 5 to <10, and ≥10).

### Statistical Analysis

We conducted data analyses from September 14 to December 2, 2020. We calculated mean (SD) values for continuous variables and percentages for categorical variables. The differences between children with and children without ADHD symptoms were analyzed using *t* tests for continuous variables and *χ*^2^ tests for categorical variables.

We analyzed the associations of ADHD symptoms and subtypes with SHS exposure from pregnancy to childhood, the timing of the SHS exposure, and current paternal smoking by fitting generalized linear mixed models with a logit link function. We fit crude models with school as the random intercept. We fit adjusted models for each outcome, adjusting for covariates collected from the questionnaires (child age, sex, only child, preterm birth and low birth weight, parental educational levels, yearly household income, maternal age, current and prenatal maternal smoking, and prenatal maternal alcohol consumption). We adjusted for previous SHS exposure in the analyses of the timing of the SHS exposure.

We conducted several sensitivity analyses: (1) we redefined ADHD symptoms using the Conners Hyperkinesis Index; (2) we redefined ADHD symptoms using the *DSM-5* criteria; (3) we analyzed the ADHD symptoms as continuous variables (the total score, the inattention and the hyperactivity-impulsivity subscores of the SIS-ADHD, and the raw score and *z* score from the Conners Hyperkinesis Index^20^); and (4) we grouped SHS exposure from pregnancy to childhood into more detailed categories of exposure (never, only current, only postnatal, only postnatal and current, only prenatal, only prenatal and current, only prenatal and postnatal, and always). No stratified analyses were conducted because the interaction terms between each exposure variable and the covariates were not significant in any analysis.

Statistical analyses were conducted with the statistical software R, version 3.6.1 (R Core Team 2019). We present the results as odds ratios (ORs) with 95% CIs. All applicable tests were 2-sided tests, and *P* < .05 was considered statistically significant.

## Results

### Characteristics of the Study Population

A total of 48 612 eligible children and adolescents participated in this survey, of which 45 562 completed the assessments of ADHD symptoms and were included in the final analyses (mean [SD] age, 11.0 [2.6] years; 50.3% girls). As shown in Table 1, 2170 children (4.8%) had ADHD symptoms. Compared with children without ADHD symptoms, children with ADHD symptoms were more likely to be older (mean [SD] age, 11.2 [2.6] vs 11.0 [2.6] years), boys (63.6% vs 49.0%), not an only child (15.5% vs 13.9%), and a preterm birth (7.1% vs 5.1%). There were differences between children with and children without ADHD symptoms in parental educational levels, yearly household income, maternal age during pregnancy, current and prenatal maternal smoking, and prenatal maternal alcohol consumption. As shown in Table 2, the prevalence of children with ADHD-HI symptoms was 0.2% (n = 91), 4.0% (n = 1816) for ADHD-I symptoms, and 0.6% (n = 263) for ADHD-C symptoms. The prevalence of having ADHD symptoms and subtypes differed across the SHS exposure categories.

**Table 1.** Demographic Characteristics of 45 562 Study Participants

Characteristic	No. (%) of participants			*P* value
	Overall	Children with ADHD symptoms (n = 2170)^a^	Children without ADHD symptoms (n = 43 392)^a^	
Child age, mean (SD), y	11.0 (2.6)	11.2 (2.6)	11.0 (2.6)	<.01
Child sex				
Boy	22 657 (49.7)	1381 (63.6)	21 276 (49.0)	<.001
Girl	22 905 (50.3)	789 (36.4)	22 116 (51.0)	
Only child				
Yes	39 212 (86.1)	1834 (84.5)	37 378 (86.1)	.03
No	6350 (13.9)	336 (15.5)	6014 (13.9)	
Preterm birth				
Yes	2349 (5.2)	153 (7.1)	2196 (5.1)	<.001
No	43 213 (94.8)	2017 (92.9)	41 196 (94.9)	
Low birth weight				
Yes	1777 (3.9)	100 (4.6)	1677 (3.9)	.08
No	43 785 (96.1)	2070 (95.4)	41 715 (96.1)	
Parental educational levels				
Low	29 113 (63.9)	1583 (72.9)	27 530 (63.4)	<.001
High	16 449 (36.1)	587 (27.1)	15 862 (36.6)	
Yearly household income, yuan				
≤10 000	10 117 (22.2)	585 (27.0)	9532 (22.0)	<.001
10 001 to 30 000	17 817 (39.1)	884 (40.7)	16 933 (39.0)	
30 001 to 100 000	15 346 (33.7)	615 (28.3)	14 731 (33.9)	
>100 000	2282 (5.0)	86 (4.0)	2196 (5.1)	
Maternal age, y				
≤25	21 093 (46.3)	1053 (48.5)	20 040 (46.2)	.01
>25 to 30	20 376 (44.7)	904 (41.7)	19 472 (44.9)	
>30 to 35	3002 (6.6)	148 (6.8)	2854 (6.6)	
>35	1091 (2.4)	65 (3.0)	1026 (2.4)	
Current maternal smoking				
Yes	564 (1.2)	61 (2.8)	503 (1.2)	<.001
No	44 998 (98.8)	2109 (97.2)	42 889 (98.8)	
Prenatal maternal smoking				
Yes	286 (0.6)	29 (1.3)	257 (0.6)	<.001
No	45 276 (99.4)	2141 (98.7)	43 135 (99.4)	
Prenatal maternal alcohol consumption				
Yes	340 (0.7)	35 (1.6)	305 (0.7)	<.001
No	45 222 (99.3)	2135 (98.4)	43 087 (99.3)	

Abbreviation: ADHD, attention-deficit/hyperactivity disorder.

^a^ The Chinese version of the Symptom Inventory Scale of ADHD consists of 18 queries categorized as 9 symptoms of inattention and 9 symptoms of hyperactivity-impulsivity. Each item or symptom was rated on a 4-point Likert scale (0, never or rare; 1, sometimes; 2, often; and 3, very often). Children with ADHD symptoms were defined as presenting often or very often with 6 or more symptoms of inattention, 6 or more symptoms of hyperactivity-impulsivity, or both.

**Table 2.** Prevalence of ADHD Symptoms and Subtypes Associated With the Timing of Secondhand Smoke Exposure Among School-Aged Children (6-18 Years)^a^

Secondhand smoke exposure	No.	No. (%) of children			
		ADHD symptoms	Subtypes of ADHD symptoms		
			ADHD-HI	ADHD-I	ADHD-C
Total	45 562	2170 (4.8)	91 (0.2)	1816 (4.0)	263 (0.6)
From birth to childhood					
Unexposed	24 642	838 (3.4)	31 (0.1)	712 (2.9)	95 (0.4)
Ever exposed	16 258	868 (5.3)	45 (0.3)	711 (4.4)	112 (0.7)
Always exposed	4662	464 (10.0)	15 (0.3)	393 (8.4)	56 (1.2)
Exposure window					
Prenatal					
No	37 337	1440 (3.9)	62 (0.2)	1200 (3.2)	178 (0.5)
Yes	8225	730 (8.9)	29 (0.4)	616 (7.5)	85 (1.0)
Postnatal					
No	36 700	422 (3.9)	57 (0.2)	1200 (3.3)	165 (0.4)
Yes	8862	748 (8.4)	34 (0.4)	616 (7.0)	98 (1.1)
Current					
No	27 902	1076 (3.9)	45 (0.2)	907 (3.3)	124 (0.4)
Yes	17 660	1094 (6.2)	46 (0.3)	909 (5.1)	139 (0.8)
Current paternal smoking, No. of cigarettes/d					
Weekdays					
0	27 200	1081 (4.0)	40 (0.1)	912 (3.4)	129 (0.5)
1 to <5	8027	414 (5.2)	19 (0.2)	347 (4.3)	48 (0.6)
5 to <10	5168	333 (6.4)	13 (0.3)	276 (5.3)	44 (0.9)
≥10	5167	342 (6.6)	19 (0.4)	281 (5.4)	42 (0.8)
Weekends					
0	27 200	1081 (4.0)	40 (0.1)	912 (3.4)	129 (0.5)
1 to <5	10 272	579 (5.6)	26 (0.3)	483 (4.7)	70 (0.7)
5 to <10	2822	181 (6.4)	11 (0.4)	150 (5.3)	20 (0.7)
≥10	5268	329 (6.2)	14 (0.3)	271 (5.1)	44 (0.8)

Abbreviations: ADHD, attention-deficit/hyperactivity disorder; ADHD-C, ADHD with combined symptoms of both inattention and hyperactivity-impulsivity; ADHD-HI, ADHD with symptoms of hyperactivity-impulsivity; ADHD-I, ADHD with symptoms of inattention.

^a^ The Chinese version of the symptom inventory scale of ADHD consists of 18 queries categorized as 9 symptoms of inattention and 9 symptoms of hyperactivity-impulsivity. Each item or symptom was rated on a 4-point Likert scale (0, never or rare; 1, sometimes; 2, often; and 3, very often). Children with ADHD symptoms were defined as presenting often or very often with 6 or more symptoms of inattention, 6 or more symptoms of hyperactivity-impulsivity, or both. The ADHD symptoms were further classified into 3 subtypes: ADHD-I, defined as presenting often or very often with 6 or more symptoms of inattention; ADHD-HI, defined as presenting often or very often with 6 or more symptoms of hyperactivity-impulsivity; and ADHD-C, defined as presenting often or very often with 6 or more symptoms of both inattention and 6 or more symptoms of hyperactivity-impulsivity.

### Associations Between SHS Exposure and ADHD Symptoms

In the fully adjusted model (Table 3), compared with their unexposed counterparts, children ever exposed to SHS (OR, 1.50; 95% CI, 1.36-1.66) or always exposed to SHS (OR, 2.88; 95% CI, 2.55-3.25) had higher odds of ADHD symptoms and higher odds of ADHD subtypes (ORs ranged from 1.46 [95% CI, 1.31-1.62] to 2.94 [95% CI, 2.09-4.13]).

**Table 3.** Associations of Secondhand Smoke Exposure From Pregnancy to Childhood With ADHD Symptoms and Subtypes Among School-Aged Children (6-18 Years)^a^

ADHD symptom or subtype	Model 1^b^		Model 2^c^	
	OR (95% CI)	*P* value	OR (95% CI)	*P* value
ADHD symptom				
Unexposed	1 [Reference]		1 [Reference]	
Ever exposed	1.59 (1.44-1.75)	<.001	1.50 (1.36-1.66)	<.001
Always exposed	3.10 (2.75-3.49)	<.001	2.88 (2.55-3.25)	<.001
ADHD-HI subtype				
Unexposed	1 [Reference]		1 [Reference]	
Ever exposed	2.22 (1.40-3.52)	<.001	2.07 (1.30-3.30)	<.001
Always exposed	2.76 (1.49-5.13)	<.001	2.42 (1.27-4.59)	.01
ADHD-I subtype				
Unexposed	1 [Reference]		1 [Reference]	
Ever exposed	1.53 (1.38-1.70)	<.001	1.46 (1.31-1.62)	<.001
Always exposed	3.09 (2.72-3.51)	<.001	2.89 (2.53-3.29)	<.001
ADHD-C subtype				
Unexposed	1 [Reference]		1 [Reference]	
Ever exposed	1.81 (1.38-2.38)	<.001	1.65 (1.25-2.18)	<.001
Always exposed	3.31 (2.38-4.62)	<.001	2.94 (2.09-4.13)	<.001

Abbreviations: ADHD, attention-deficit/hyperactivity disorder; ADHD-C, ADHD with combined symptoms of both inattention and hyperactivity-impulsivity; ADHD-HI, ADHD with symptoms of hyperactivity-impulsivity; ADHD-I, ADHD with symptoms of inattention; OR, odds ratio.

^a^ The Chinese version of the symptom inventory scale of ADHD consists of 18 queries categorized as 9 symptoms of inattention and 9 symptoms of hyperactivity-impulsivity. Each item or symptom was rated on a 4-point Likert scale (0, never or rare; 1, sometimes; 2, often; and 3, very often). Children with ADHD symptoms were defined as presenting often or very often with 6 or more symptoms of inattention, 6 or more symptoms of hyperactivity-impulsivity, or both. The ADHD symptoms were further classified into 3 subtypes: ADHD-I, defined as presenting often or very often with 6 or more symptoms of inattention; ADHD-HI, defined as presenting often or very often with 6 or more symptoms of hyperactivity-impulsivity; and ADHD-C, defined as presenting often or very often with both 6 or more symptoms of inattention and 6 or more symptoms of hyperactivity-impulsivity.

^b^ Adjusted for a school-level random intercept.

^c^ Further adjusted for child age, sex, only child, preterm birth and low birth weight, parental educational levels, yearly household income, maternal age, current and prenatal maternal smoking, and prenatal maternal alcohol consumption.

In the fully adjusted model (Table 4), compared with their unexposed counterparts, children with SHS exposure had higher odds of having ADHD symptoms if they were exposed during the prenatal period (OR, 2.28; 95% CI, 2.07-2.51), early postnatal period (OR, 1.47; 95% CI, 1.29-168), or current period (OR, 1.20; 95% CI, 1.09-1.31). Both prenatal SHS exposure and early postnatal SHS exposure were associated with all ADHD subtypes (ORs ranged from 1.38 [95% CI, 1.20-1.59] to 2.32 [95% CI, 2.09-2.57]), whereas current SHS exposure was associated only with the ADHD-I subtype (OR, 1.19; 95% CI, 1.07-1.32).

**Table 4.** Associations of the Timing of SHS Exposure With ADHD Symptoms and Subtypes Among School-Aged Children (6-18 Years)^a^

Exposure window of SHS	Model 1^b^		Model 2^c^	
	OR (95% CI)	*P* value	OR (95% CI)	*P* value
Prenatal (reference: prenatal unexposed)				
ADHD symptom	2.40 (2.18-2.63)	<.001	2.28 (2.07-2.51)	<.001
ADHD-HI subtype	2.24 (1.44-3.49)	<.001	2.06 (1.30-3.25)	<.001
ADHD-I subtype	2.43 (2.20-2.69)	<.001	2.32 (2.09-2.57)	<.001
ADHD-C subtype	2.27 (1.75-2.94)	<.001	2.08 (1.59-2.71)	<.001
Postnatal (reference: postnatal unexposed)				
ADHD symptom	2.26 (2.06-2.48)	<.001	1.47 (1.29-1.68)	<.001
ADHD-HI subtype	2.62 (1.71-4.01)	<.001	2.11 (1.16-3.81)	.01
ADHD-I subtype	2.21 (2.00-2.44)	<.001	1.38 (1.20-1.59)	<.001
ADHD-C subtype	2.56 (1.99-3.29)	<.001	1.98 (1.39-2.81)	<.001
Current (reference: current unexposed)				
ADHD symptom	1.63 (1.49-1.77)	<.001	1.20 (1.09-1.31)	<.001
ADHD-HI subtype	1.64 (1.08-2.47)	.02	1.13 (0.72-1.79)	.60
ADHD-I subtype	1.61 (1.46-1.77)	<.001	1.19 (1.07-1.32)	<.001
ADHD-C subtype	1.80 (1.42-2.30)	<.001	1.28 (0.98-1.67)	.07

Abbreviations: ADHD, attention-deficit/hyperactivity disorder; ADHD-C, ADHD with combined symptoms of inattention and hyperactivity-impulsivity; ADHD-HI, ADHD with symptoms of hyperactivity-impulsivity; ADHD-I, ADHD with symptoms of inattention; OR, odds ratio; SHS, secondhand smoke.

^a^ The Chinese version of the symptom inventory scale of ADHD consists of 18 queries categorized as 9 symptoms of inattention and 9 symptoms of hyperactivity-impulsivity. Each item or symptom was rated on a 4-point Likert scale (0, never or rare; 1, sometimes; 2, often; and 3, very often). Children with ADHD symptoms were defined as presenting often or very often with 6 or more symptoms of inattention, 6 or more symptoms of hyperactivity-impulsivity, or both. The ADHD symptoms were further classified into 3 subtypes: ADHD-I, defined as presenting often or very often with 6 or more symptoms of inattention; ADHD-HI, defined as presenting often or very often with 6 or more symptoms of hyperactivity-impulsivity; and ADHD-C, defined as presenting often or very often with 6 or more symptoms of both inattention and 6 or more symptoms of hyperactivity-impulsivity.

^b^ Adjusted for a school-level random intercept.

^c^ Further adjusted for child age, sex, only child, preterm birth and low birth weight, parental educational levels, yearly household income, maternal age, current and prenatal maternal smoking, prenatal maternal alcohol consumption, and previous SHS exposure.

Further investigation of current paternal cigarette smoking (Table 5) indicated that, compared with their unexposed counterparts, children whose fathers smoked 10 or more cigarettes/d on both weekdays and weekends had higher odds of having ADHD symptoms and subtypes (ORs ranged from 1.48 [95% CI, 1.28-1.70] to 2.25 [95% CI, 1.29-3.93]) except for the association between weekday paternal cigarette smoking and ADHD-HI symptoms. Compared with their unexposed counterparts, children with fathers who smoked cigarettes had higher odds of ADHD-HI subtype (OR, 2.41; 95% CI, 1.22-4.76) only when their fathers smoked 5 to <10 cigarettes/d on weekdays.

**Table 5.** Associations of Current Paternal Smoking and ADHD Symptoms and Subtypes Among School-Aged Children (6-18 Years)^a^^,^^b^

No. of cigarettes per day	Weekdays		Weekends	
	OR (95% CI)	*P* value	OR (95% CI)	*P* value
ADHD symptom				
0	1 [Reference]		1 [Reference]	
1 to <5	1.35 (1.21-1.50)	<.001	1.24 (1.11-1.40)	<.001
5 to <10	1.52 (1.29-1.80)	<.001	1.53 (1.35-1.74)	<.001
≥10	1.50 (1.32-1.70)	<.001	1.57 (1.39-1.79)	<.001
ADHD-HI subtype				
0	1 [Reference]		1 [Reference]	
1 to <5	1.63 (0.99-2.70)	.06	1.53 (0.88-2.66)	.13
5 to <10	2.41 (1.22-4.76)	.01	1.56 (0.83-2.95)	.17
≥10	1.61 (0.87-2.99)	.13	2.25 (1.29-3.93)	<.001
ADHD-I subtype				
0	1 [Reference]		1 [Reference]	
1 to <5	1.34 (1.19-1.50)	<.001	1.24 (1.09-1.41)	<.001
5 to <10	1.51 (1.26-1.80)	<.001	1.52 (1.32-1.75)	<.001
≥10	1.48 (1.28-1.70)	<.001	1.55 (1.35-1.78)	<.001
ADHD-C subtype				
0	1 [Reference]		1 [Reference]	
1 to <5	1.34 (0.99-1.80)	.05	1.19 (0.85-1.66)	.31
5 to <10	1.36 (0.84-2.19)	.21	1.64 (1.16-2.32)	.01
≥10	1.62 (1.15-2.30)	.01	1.57 (1.10-2.24)	.01

Abbreviations: ADHD, attention-deficit/hyperactivity disorder; ADHD-C, ADHD with combined symptoms of inattention and hyperactivity-impulsivity; ADHD-HI, ADHD with symptoms of hyperactivity-impulsivity; ADHD-I, ADHD with symptoms of inattention; OR, odds ratio.

^a^ The Chinese version of the symptom inventory scale of ADHD consists of 18 queries categorized as 9 symptoms of inattention and 9 symptoms of hyperactivity-impulsivity. Each item or symptom was rated on a 4-point Likert scale (0, never or rare; 1, sometimes; 2, often; and 3, very often). Children with ADHD symptoms were defined as presenting often or very often with 6 or more symptoms of inattention or 6 or more symptoms of hyperactivity-impulsivity, or both. The ADHD symptoms were further classified into 3 subtypes: ADHD-I, defined as presenting often or very often with 6 or more symptoms of inattention; ADHD-HI, defined as presenting often or very often with 6 or more symptoms of hyperactivity-impulsivity; and ADHD-C, defined as presenting often or very often with both 6 or more symptoms of inattention and 6 or more symptoms of hyperactivity-impulsivity.

^b^ Results are shown using the adjusted models with the adjustment of school-level random intercepts, child age, sex, only child, preterm birth and low birth weight, parental educational levels, yearly household income, maternal age, current and prenatal maternal smoking, and prenatal maternal alcohol consumption.

### Sensitivity Analyses

When we redefined ADHD symptoms using the Conners Hyperkinesis Index, the results indicated only minor changes in the adjusted models except that the association between current SHS exposure and ADHD symptoms attenuated to the null (eTable 2 in the Supplement). When we redefined ADHD symptoms using the *DSM-5* criteria or using continuous scores from the SIS-ADHD scale or the Conners Hyperkinesis Index, the associations remained robust in all analyses (eTables 3-8 in the Supplement). When grouping the SHS exposure from pregnancy to childhood into more detailed categories (eTable 9 in the Supplement), children in all categories had higher odds of having ADHD or ADHD-I symptoms compared with their unexposed counterparts (ORs ranged from 1.21 [95% CI, 1.06-1.38] to 2.90 [95% CI, 2.54-3.30]), whereas the associations of ADHD-HI and ADHD-C symptoms attenuated to the null in several categories (ie, only current exposure, only prenatal exposure, and only prenatal and current exposure).

## Discussion

In this large cross-sectional study, we found that SHS exposure from pregnancy to childhood was associated with ADHD symptoms and subtypes in school-aged children. When considering the timing of the SHS exposure, the associations were more pronounced in the prenatal and early postnatal periods. In addition, children with fathers who smoked cigarettes had higher odds of having ADHD symptoms and subtypes.

Inconsistent results were previously obtained in a meta-analysis assessing prenatal SHS exposure in association with ADHD symptoms.^21^ By contrast, a recently published systematic review reported that postnatal SHS exposure was associated with ADHD symptoms (combined OR, 1.61; 95% CI, 1.37-1.88).^22^ When combining SHS exposures from the prenatal and postnatal periods, previous studies and our results indicated that the associations may be stronger. Three cohorts (2 in Germany^11,12^ and 1 in Hong Kong, China^10^) indicated that SHS exposure from pregnancy to childhood was associated with ADHD symptoms in children 10 years of age based on rating scales without considering ADHD subtypes. A case-control study suggested that SHS exposure in both prenatal and early postnatal periods was associated with ADHD and the ADHD-HI subtype based on clinical interviews with Korean children 6 to 10 years of age.^6^ The most rapid period of brain growth with the highest plasticity occurs in the last trimester of pregnancy and the first 2 years of life (ie, the early postnatal period), providing the time of greatest brain vulnerability to any SHS exposure.^9^ However, late postnatal SHS exposure may also be important because genetic and neurobiological associations with ADHD-related symptoms are enhanced during that period.^5,6,23,24,25,26,27,28^ Cotinine, the metabolite of nicotine contained in SHS smoke, has been commonly used to determine current SHS exposure when studying the role of late postnatal SHS exposure. For example, the performance on an attention test has been correlated with urine cotinine levels in Egyptian children (aged 10-12 years).^29^ In addition, 2 case-control studies in Korea^6,26^ and 2 cross-sectional studies in the US^23,28^ suggested that urine or serum cotinine levels were associated with higher parental self-reported or diagnosed ADHD symptoms in school-aged children across different age groups. Moreover, a 3-year follow-up study in Hong Kong, China, indicated that saliva cotinine levels were associated with ADHD symptoms based on rating scales.^30^ In our study, although the association between current SHS exposure and ADHD symptoms was less pronounced, we believe that the focus should not only be on SHS exposure in the first 1000 days of life; effective and continuous interventions for parental smoking cessation in late postnatal periods are still needed.

Mechanistically, neuronal nicotinic acetylcholine receptors (nAChRs), which are primary targets for unhealthy activation by nicotine exposure, regulate brain maturation.^31^ Rodent studies indicate that developmental nicotine exposure induces abnormal expression of nAChRs, hypersensitivity to nAChR-mediated dopamine release, and dopamine transporter dysfunction in the frontal cortex.^32^ The regulation of attentional behaviors by the prefrontal cortex is dependent on optimal levels of dopamine,^33^ and a functional reduction of dopamine in the prefrontal cortex may lead to ADHD-related symptoms.^34^ Neuroimaging studies indicate that young adults prenatally exposed to nicotine exhibit weaker activation of the inferior frontal gyrus during attention test paradigms than their unexposed counterparts, suggesting a functional involvement of prenatal exposure to tobacco smoke in ADHD-related neural alterations.^35^ Because the expression of neural nAChRs continues through gestation into adulthood, the developmental defects caused by nicotine may be dependent on the timing of exposure. Therefore, more studies are needed to understand the association of SHS exposure from pregnancy to childhood with ADHD symptoms.

It is important to encourage all adults to avoid smoking, and our results indicated that fathers may be one of the most important target populations to reduce SHS exposure. There is still limited evidence of effective interventions for reducing SHS exposure in children.^36^ Pregnancy is considered a reasonable time to help parents who smoke cigarettes to quit. However, few studies have targeted expectant fathers,^14^ and current evidence suggests that the behavioral interventions for smoking relapse after birth are not effective.^37^ Both fathers and mothers need to be informed of the neurotoxic effects associated with SHS exposure throughout the development of children, and more interventions for smoking cessation are needed.

Among the ADHD subtypes, 2 case-control studies in Korea found that current SHS exposure (ie, cotinine level) was associated with the ADHD-I subtype,^6,26^ whereas early postnatal SHS exposure was associated with the ADHD-HI subtype.^6^ No previous study has investigated the association between the ADHD-C subtype and exposure to SHS. In our study, the association between SHS exposure and ADHD-I symptoms was more pronounced than the other 2 subtypes, but these results should be interpreted cautiously. The prevalence of ADHD symptoms observed in our study was slightly lower than that observed in a meta-analyses of Chinese studies, and the relative proportion of subtypes was also different compared with other areas of China.^38^ The methodological approaches, including the sampling process, sources of information, and ADHD-related measurements, may have contributed to these differences. In addition, the small number of children with the ADHD-HI and ADHD-C subtypes may have decreased the statistical power of our study. Future research is needed to understand the heterogeneity regarding the association between SHS exposure and different ADHD subtypes.

### Strengths and Limitations

Several study limitations should be taken into consideration. First, because this was a cross-sectional study, we were unable to assess the temporality of our results. Second, we used self-reported questionnaires to measure SHS exposure, which may have resulted in recall bias and exposure misclassification. However, a previous review indicated that it is the most cost-effective method to assess long-term SHS exposure for different critical developmental periods in pediatric research.^39^ Third, although we used validated *DSM-IV*–based questionnaires to identify ADHD symptoms and subtypes in our study, we were unable to diagnose the cases. However, our results were robust across different sensitivity analyses. Fourth, we did not collect data on parental ADHD history, and therefore we cannot exclude the possibility that the associations may be confounded by parental ADHD history. Despite these limitations, our study had notable strengths, including a large sample size covering a wide developmental range of children, comprehensive information on SHS exposure with numerous exposure windows, and several ADHD assessments, all which helped to strengthen our findings.

## Conclusions

Our study results indicated that SHS exposure from pregnancy to childhood was associated with higher odds of ADHD symptoms and subtypes in school-aged children, with somewhat stronger associations observed for prenatal and early postnatal periods. Our findings highlight the importance of strengthening public health efforts to reduce SHS exposure, which may reduce the health and economic burdens of individuals with ADHD.

## Notes

Supplement. **eTable 1.** Summaries of Previous Observational Studies Regarding the Associations of SHS Exposure With ADHD Symptoms and Subtypes in Children Population **eTable 2.** The Associations of SHS Exposure With ADHD Symptoms and Subtypes in School-Aged Children (6-18 Years) Using the Cutoff of C-ASQ **eTable 3.** The Associations of SHS Exposure From Pregnancy to Childhood With ADHD Symptoms and Subtypes in School-Aged Children (6-18 Years) Using DSM-5 Criteria **eTable 4.** The Associations of SHS Exposure With ADHD Symptoms and Subtypes In Different Timing In School-Aged Children (6-18 Years) Using DSM-5 Criteria **eTable 5.** The Associations of Current Paternal Smoking and ADHD Symptoms and Subtypes in School-Aged Children (6-18 Years) Using DSM-5 Criteria **eTable 6.** The Associations of SHS Exposure From Pregnancy to Childhood With ADHD Symptoms and Subtypes in School-Aged Children (6-18 Years) Using the Continuous Scores of the Symptom Inventory Scale of ADHD and C-ASQ **eTable 7.** The Associations of SHS Exposure With ADHD Symptoms and Subtypes in Different Timing in School-Aged Children (6-18 Years) Using the Continuous Scores of the Symptom Inventory Scale of ADHD and C-ASQ **eTable 8.** The Associations of Current Paternal Smoking and ADHD Symptoms and Subtypes in School-Aged Children (6-18 Years) Using the Continuous Scores of the Symptom Inventory Scale of ADHD and C-ASQ **eTable 9.** The Associations of SHS Exposure in Different Timing With ADHD Symptom and Subtypes in School-Aged Children (6-18 Years)
