# Supplementary material for: Association of Prenatal, Early Postnatal, or Current Exposure to Secondhand Smoke With Attention-Deficit/Hyperactivity Disorder Symptoms in Children
Source: JAMA Netw Open. 2021 May 20;4(5):e2110931. doi: 10.1001/jamanetworkopen.2021.10931 (PMC8138689; doi:10.1001/jamanetworkopen.2021.10931)
Supplement: Supplement. — eTable 1. Summaries of Previous Observational Studies Regarding the Associations of SHS Exposure With ADHD Symptoms and Subtypes in Children Population eTable 2. The Associations of SHS Exposure With ADHD Symptoms and Subtypes in School-Aged Children (6-18 Years) Using the Cutoff of C-ASQ eTable 3. The Associations of SHS Exposure From Pregnancy to Childhood With ADHD Symptoms and Subtypes in School-Aged Children (6-18 Years) Using DSM-5 Criteria eTable 4. The Associations of SHS Exposure With ADHD Symptoms and Subtypes In Different Timing In School-Aged Children (6-18 Years) Using DSM-5 Criteria eTable 5. The Associations of Current Paternal Smoking and ADHD Symptoms and Subtypes in School-Aged Children (6-18 Years) Using DSM-5 Criteria eTable 6. The Associations of SHS Exposure From Pregnancy to Childhood With ADHD Symptoms and Subtypes in School-Aged Children (6-18 Years) Using the Continuous Scores of the Symptom Inventory Scale of ADHD and C-ASQ eTable 7. The Associations of SHS Exposure With ADHD Symptoms and Subtypes in Different Timing in School-Aged Children (6-18 Years) Using the Continuous Scores of the Symptom Inventory Scale of ADHD and C-ASQ eTable 8. The Associations of Current Paternal Smoking and ADHD Symptoms and Subtypes in School-Aged Children (6-18 Years) Using the Continuous Scores of the Symptom Inventory Scale of ADHD and C-ASQ eTable 9. The Associations of SHS Exposure in Different Timing With ADHD Symptom and Subtypes in School-Aged Children (6-18 Years) [file jamanetwopen-e2110931-s001.pdf]

Supplementary Online Content

Lin LZ, Xu SL, Wu QZ, et al. Association of prenatal, early postnatal, or current exposure to secondhand smoke with attention-deficit/hyperactivity disorder symptoms in children. *JAMA Netw Open.* 2021;4(5):e2110931. doi:10.1001/jamanetworkopen.2021.10931

eTable 1. Summaries of Previous Observational Studies Regarding the Associations of SHS Exposure With ADHD Symptoms and Subtypes in Children Population
eTable 2. The Associations of SHS Exposure With ADHD Symptoms and Subtypes in School-Aged Children (6-18 Years) Using the Cutoff of C-ASQ
eTable 3. The Associations of SHS Exposure From Pregnancy to Childhood With ADHD Symptoms and Subtypes in School-Aged Children (6-18 Years) Using DSM-5 Criteria
eTable 4. The Associations of SHS Exposure With ADHD Symptoms and Subtypes In Different Timing In School-Aged Children (6-18 Years) Using DSM-5 Criteria
eTable 5. The Associations of Current Paternal Smoking and ADHD Symptoms and Subtypes in School-Aged Children (6-18 Years) Using DSM-5 Criteria
eTable 6. The Associations of SHS Exposure From Pregnancy to Childhood With ADHD Symptoms and Subtypes in School-Aged Children (6-18 Years) Using the Continuous Scores of the Symptom Inventory Scale of ADHD and C-ASQ
eTable 7. The Associations of SHS Exposure With ADHD Symptoms and Subtypes in Different Timing in School-Aged Children (6-18 Years) Using the Continuous Scores of the Symptom Inventory Scale of ADHD and C-ASQ
eTable 8. The Associations of Current Paternal Smoking and ADHD Symptoms and Subtypes in School-Aged Children (6-18 Years) Using the Continuous Scores of the Symptom Inventory Scale of ADHD and C-ASQ
eTable 9. The Associations of SHS Exposure in Different Timing With ADHD Symptom and Subtypes in School-Aged Children (6-18 Years)

This supplementary material has been provided by the authors to give readers additional information about their work.

© 2021 Lin LZ et al. *JAMA Network Open*.

eTable 1. Summaries of Previous Observational Studies Regarding the Associations of SHS Exposure With ADHD Symptoms and Subtypes in Children Population

Author	Study Period	Sample Size	Location	Outcomes	Exposure		Main Findings [a]
					Measurements	Types	
**Cross-sectional study**							
Mahabee-Gittens (2020)	2016-2017	21,539 (6-11 yrs)	The USA	Parental report	Questionnaires (Current)	no exposure	Ref
						household smoker with no home exposure	**1.44 (1.27−1.63)**
						household smoker with home exposure	**1.65 (1.24−2.19)**
Wang (2019)	2014.6	15,344 (9 yrs)	Shanghai, China	Rating scale (SDQ)	Questionnaires (Current)	None	**Ref**
						< 1 h/daily	**1.08 (0.14, 2.01)**
						1–2 h/daily	**1.18 (0.20, 2.16)**
						⩾3 h/daily	**1.02 (0.06, 1.99)**
Padrón (2015)	2011-2012	2357 (4-12 yrs)	Spain	Rating scale (SDQ)	Questionnaires (Current)	not habitually exposed	Ref
						<1 h/day	**2.18 (1.30−3.64)**
						≥1 h/day	**3.14 (1.63−6.04)**
Shiue (2015)	2013	1,019 (4-12 yrs)	Scotland	Rating scale (SDQ)	Questionnaires (Current)	Not allowed	Ref
						Anywhere	**3.86 (1.17–12.72)**
						Some areas	**2.85 (1.34–6.05)**
						Outdoor areas	1.78 (0.92–3.50)
Bauer (2014)	2004.11-2012-6	2,441 (0-6 yrs)	Indianapolis, the USA	Electronic health record (ICD-9）	Questionnaires (Current)	Yes (Ref: No)	**1.9 (1.2–3.1)**
Max (2013)	1999-2004	6283/6033 (4-15 yrs)	Seattle, the USA	Parental report	Questionnaires (Current)	Yes (Ref: No)	**1.5 (1.1–2.0)**
					Serum cotinine (Current)	Yes (Ref: No)	**1.8 (1.3–2.5)**
Kabir (2011)	2007.4-2008.7	55,358 (0-11 yrs)	The USA	Parental report	Questionnaires (Current)	Yes (Ref: No)	**1.44 (1.21–1.72)**
Twardella (2010)	2005-2006	5,494 (5-7 yrs)	Bavaria, Germany	Rating scale (SDQ)	Questionnaires (Postnatal)	Yes (Ref: No)	**2.39 (1.62–3.53)**
Bandiera (2011)	2001-2004	2,901 (8-15 yrs)	The USA	Rating scales (DSM-IV)	Serum cotinine (Current)	NA	**0.40 (0.24–0.56)**[a]
				Diagnosis (DSM-IV)			1.03 (0.92–1.16)
Braun (2006)	1999-2002	3,879 (4015 yrs)	The USA	Parental report	Questionnaires (Prenatal)	Yes (Ref: No)	**2.5 (1.2–5.2)**
					Questionnaires (Postnatal)	Yes (Ref: No)	0.6 (0.3–1.3)
**Case- control study**							
Kim (2018)	2017.1-2017.12	Case: 200 Control: 253 (6-9 yrs)	Cheonan,Korea	Diagnosis (DSM-IV)	Urine cotinine (Current)	NA	**1.55 (1.05−2.30)**
Joo	2008-	Case: 214	Cheonan,Korea	Diagnosis	Urine cotinine (Current)	NA	**1.10 (1.03–1.16)**

© 2021 Lin LZ et al. *JAMA Network Open*.

(2017)	2010	Control: 214 (6-10 yrs)		(DSM-IV)	Questionnaires (Prenatal)	Yes (Ref: No)	**1.67 (1.08–2.58)**
					Questionnaires (Postnatal)	Yes (Ref: No)	**1.83 (1.15-2.91)**
					Questionnaires (Pre-and postnatal)	Both yes (Ref: Both No)	**2.41 (1.20–4.81)**
Yoshimasu (2009)	2006.3-2008.12	Case: 90 Control: 270 (6-15 yrs)	Kinki, Japan	Diagnosis (DSM-IV)	Questionnaires (Prenatal)	Yes (Ref: No)	1.0 (0.4–2.3)
**Cohort study**							
Minatoya (2019)	2008.4-2017.5	3,216 (5 yrs)	Hokkaido, Japan	Rating scale (SDQ)	Blood cotinine (Prenatal)	≤ 0.21 ng/ml	Ref
						0.22–0.51 ng/ml (low passive)	1.23 (0.89–1.69)
						0.52–11.48 ng/ml (high passive)	1.15 (0.82–1.60)
						≧ 11.49 ng/ml (active)	1.49 (1.00–2.23)
Downey (2015)	2002-2004	826 (2 yrs)	the USA	Rating scale (CBCL and ADHD[P])	Questionnaires (Prenatal)	Yes (Ref: No)	**1.9 (1.2–3.2)**
						Yes (Ref: No)	**2.4 (1.4–4.2)**
Leung (2015)	1997.4-2012.8	7,914 (7-12 yrs)	Hong Kong, China	Rating scale (RPRS)	Questionnaires (Different period)	No prenatal and no postnatal exposure	Ref
						Only occasional prenatal exposure from non-parental sources	0.07 (-0.04–0.17)
						Only daily prenatal exposure from non–parental sources	0.04 (-0.21–0.30)
						Only postnatal exposure from non–parental sources	0.18 (-0.14–0.49)
Langley (2012)	From 1991.4.1	5,719 (7.5 yrs)	Avon, the UK	Rating scale (DAWBA)	Questionnaires (Prenatal)	Paternal smoking & nonsmoking mother (Ref: No)	**0.12 (0.04–0.20)**
					Questionnaires (Prenatal)	Household passive smoking & nonsmoking parents (Ref: No)	0.13 (−0.13–0.39)
					Questionnaires (Prenatal)	Workplace passive smoking and nonsmoking parents (Ref: No)	0.04 (−0.16–0.25)
				Diagnosis (DSM-IV)	Questionnaires (Prenatal)	Yes (Ref: No)	1.43 (0.98–2.07)
Tiesler (2011)	1997-2009	1,654 (10 yrs)	Munich, Leipzig, Wesel and Bad Honnef, Germany	Rating scale (SDQ)	Questionnaires (Different period)	Never	Ref
						Only prenatal	**1.59 (1.11–2.27)**
						Only postnatal	**1.59 (1.11–2.27)**
						Pre-and postnatal	**1.77 (1.11–2.84)**
					Questionnaires (Different period, non-smoker mothers)	Never	Ref
						Only prenatal	1.41 (0.79–2.52)
						Only postnatal	1.41 (0.79–2.52)
						Pre-and postnatal	**1.97 (1.06–3.65)**
Rückinger (2010)	1995.9-2008.7	2,862 (10 yrs)	Munich and Wesel, Germany	Rating scale (SDQ)	Questionnaires (Different period)	Never	Ref
						Only prenatal	1.1 (0.5-2.7)

© 2021 Lin LZ et al. *JAMA Network Open*.

						Only postnatal	1.2 (0.9-1.6)
						Pre-and postnatal	**1.8 (1.3-2.6)**
					Questionnaires (Prenatal)	Never	1 Ref
						Low intensity	0.4 (0.1-3.0)
						High intensity	1.9 (0.6-6.0)
Luk (2018)	2012-2015	189 (3 yrs)	Hong Kong, China	Rating scale (SDQ)	Saliva cotinine (postnatal)	NA	**1.02 (0.06, 1.99)**

Abbreviations: SHS, secondhand smoke; ADHD, attention-deficit/hyperactivity disorder; ICD-9, International Classification of Diseases Nineth Revision; DSM-IV, The Diagnostic and Statistical Manual of Mental Disorders, Fourth Edition; ADHD [p], attention problems and Diagnostic and Statistical Manual of Mental Disorders–compatible attention deficit/hyperactivity problems; CBCL, The Child Behavior Checklist; RPRS, Revised Parent's Rutter Scales; DAWBA, Development and Well-Being Assessment.

[a] The results were expressed as beta (95%CI) or odd ratios (95%CI).

© 2021 Lin LZ et al. *JAMA Network Open*.

eTable 2. The Associations of SHS Exposure With ADHD Symptoms and Subtypes in School-Aged Children (6-18 Years) Using the Cutoff of C-ASQ [a, b]

SHS exposure		ADHD (C-ASQ)	
		OR (95% CI)	P value
Prenatal exposed			
No		1 [Reference]	
Yes		2.17 (1.89, 2.48)	<0.001
Postnatal exposed			
No		1 [Reference]	
Yes		1.74 (1.45, 2.09)	<0.001
Current exposed			
No		1 [Reference]	
Yes		1.14 (0.99, 1.31)	0.06
From birth to childhood			
Unexposed		1 [Reference]	
Ever exposed		1.59 (1.38, 1.83)	<0.001
Always exposed		2.65 (2.22, 3.16)	<0.001
Current paternal smoking at weekdays (No. of cigarettes/d)			
0		1 [Reference]	
1~5		1.22 (1.05, 1.42)	0.01
5~10		1.62 (1.29, 2.02)	<0.001
≥10		1.44 (1.20, 1.74)	<0.001
Current paternal smoking at weekends (No. of cigarettes/d)			
0		1 [Reference]	
1~5		1.18 (0.99, 1.39)	0.06
5~10		1.42 (1.18, 1.72)	<0.001
≥10		1.53 (1.27, 1.83)	<0.001

Abbreviations: ADHD, attention-deficit/hyperactivity disorder; C-ASQ, Conners Abbreviated Symptom Questionnaire; CI, confidence interval; OR, odd ratios; SHS, secondhand smoke.

[a] The Conners Abbreviated Symptom Questionnaire is a set of ten checklist items rated on a 4-point Likert scale: 0 = never or rare; 1 = sometimes; 2 = often and 3 = very often. The Conners Hyperkinesis Index is a sum of the total score ranged from zero to thirty, and a score greater than fifteen was defined as having ADHD symptoms.

[b] The results were shown using the adjusted models with the adjustment of school-level random intercepts, child age, sex, only child, preterm birth and low birth weight, parental educational levels, yearly household income, maternal age, current and prenatal maternal smoking and prenatal maternal alcohol consumption.

© 2021 Lin LZ et al. *JAMA Network Open*.

eTable 3. The Associations of SHS Exposure From Pregnancy to Childhood With ADHD Symptoms and Subtypes in School-Aged Children (6-18 Years) Using DSM-5 Criteria [a, b]

ADHD symptom or subtype	Adjusted model [b]	
	OR (95% CI)	*P* value
ADHD symptom (DSM-5)		
Unexposed	1 [Reference]	
Ever exposed	1.45 (1.36, 1.54)	<0.001
Always exposed	2.50 (2.31, 2.72)	<0.001
ADHD-HI subtype (DSM-5)		
Unexposed	1 [Reference]	
Ever exposed	1.58 (1.23, 2.03)	<0.001
Always exposed	2.10 (1.47, 2.99)	<0.001
ADHD-I subtype (DSM-5)		
Unexposed	1 [Reference]	
Ever exposed	1.40 (1.31, 1.50)	<0.001
Always exposed	2.38 (2.17, 2.60)	<0.001
ADHD-C subtype (DSM-5)		
Unexposed	1 [Reference]	
Ever exposed	1.66 (1.45, 1.90)	<0.001
Always exposed	3.24 (2.73, 3.84)	<0.001

Abbreviations: ADHD, attention-deficit/hyperactivity disorder; ADHD-C, ADHD with combined symptoms of inattention and hyperactivity-impulsivity; ADHD-HI, ADHD with symptoms of hyperactivity-impulsivity; ADHD-I, ADHD with symptoms of inattention; CI, confidence interval; DSM-5, the Diagnostic and Statistical Manual of Mental Disorders-Fifth Edition; OR, odd ratios; SHS, secondhand smoke.

[a] The definition of ADHD using the DSM-5 criteria could be defined using the Chinese version of the symptom inventory scale of ADHD. Children with ADHD symptoms were defined as presenting often or very often with 5 or more symptoms of inattention, or more symptoms of hyperactivity-impulsivity, or both. The ADHD symptom were further classified into 3 subtypes: ADHD-I, defined as presenting often or very often with 5 or more symptoms of inattention; ADHD-HI, defined as presenting often or very often with 5 or more symptoms of hyperactivity-impulsivity; and ADHD-C, defined as presenting often or very often with 5 or more symptoms of both inattention and 5 or more symptoms of hyperactivity-impulsivity.

[b] The results were shown using the adjusted models with the adjustment of school-level random intercepts, child age, sex, only child, preterm birth and low birth weight, parental educational levels, yearly household income, maternal age, current and prenatal maternal smoking and prenatal maternal alcohol consumption.

© 2021 Lin LZ et al. *JAMA Network Open*.

eTable 4. The Associations of SHS Exposure With ADHD Symptoms and Subtypes In Different Timing In School-Aged Children (6-18 Years) Using DSM-5 Criteria [a, b]

Exposure windows of SHS		Adjusted model [b]	
		OR (95% CI)	*P* value
Prenatal (reference: prenatal unexposed)			
ADHD symptom (DSM-5)		2.01 (1.88, 2.14)	<0.001
ADHD-HI subtype (DSM-5)		1.55 (1.18, 2.03)	<0.001
ADHD-I subtype (DSM-5)		1.93 (1.80, 2.07)	<0.001
ADHD-C subtype (DSM-5)		2.49 (2.19, 2.84)	<0.001
Postnatal (reference: postnatal unexposed)			
ADHD symptom (DSM-5)		1.52 (1.40, 1.66)	<0.001
ADHD-HI subtype (DSM-5)		2.21 (1.59, 3.08)	<0.001
ADHD-I subtype (DSM-5)		1.48 (1.35, 1.63)	<0.001
ADHD-C subtype (DSM-5)		1.56 (1.30, 1.87)	<0.001
Current (reference: current unexposed)			
ADHD symptom (DSM-5)		1.21 (1.14, 1.28)	<0.001
ADHD-HI subtype (DSM-5)		1.27 (0.99, 1.63)	0.06
ADHD-I subtype (DSM-5)		1.20 (1.12, 1.28)	<0.001
ADHD-C subtype (DSM-5)		1.21 (1.06, 1.38)	<0.001

ADHD, attention-deficit/hyperactivity disorder; ADHD-C, ADHD with combined symptoms of inattention and hyperactivity-impulsivity; ADHD-HI, ADHD with symptoms of hyperactivity-impulsivity; ADHD-I, ADHD with symptoms of inattention; CI, confidence interval; DSM-5, the Diagnostic and Statistical Manual of Mental Disorders-Fifth Edition; OR, odd ratios; SHS, secondhand smoke.

[a] The definition of ADHD using the DSM-5 criteria could be defined using the Chinese version of the symptom inventory scale of ADHD. Children with ADHD symptoms were defined as presenting often or very often with 5 or more symptoms of inattention, or more symptoms of hyperactivity-impulsivity, or both. The ADHD symptom were further classified into 3 subtypes: ADHD-I, defined as presenting often or very often with 5 or more symptoms of inattention; ADHD-HI, defined as presenting often or very often with 5 or more symptoms of hyperactivity-impulsivity; and ADHD-C, defined as presenting often or very often with 5 or more symptoms of both inattention and 5 or more symptoms of hyperactivity-impulsivity.

[b] The results were shown using the adjusted models with the adjustment of school-level random intercepts, child age, sex, only child, preterm birth and low birth weight, parental educational levels, yearly household income, maternal age, current and prenatal maternal smoking and prenatal maternal alcohol consumption.

© 2021 Lin LZ et al. *JAMA Network Open*.

eTable 5. The Associations of Current Paternal Smoking and ADHD Symptoms and Subtypes in School-Aged Children (6-18 Years) Using DSM-5 Criteria [a, b]

Numbers of cigarettes per day		Weekdays			Weekends	
		OR (95% CI)	P Value		OR (95% CI)	P Value
ADHD symptom (DSM-5)						
0		1 [Reference]			1 [Reference]	
1 to <5		1.31 (1.22, 1.40)	<0.001		1.23 (1.15, 1.33)	<0.001
5 to <10		1.45 (1.30, 1.61)	<0.001		1.45 (1.34, 1.58)	<0.001
≥10		1.40 (1.29, 1.52)	<0.001		1.45 (1.34, 1.58)	<0.001
ADHD-HI subtype (DSM-5)						
0		1 [Reference]			1 [Reference]	
1 to <5		1.50 (1.14, 1.97)	<0.001		1.49 (1.11, 2.01)	0.01
5 to <10		1.69 (1.10, 2.59)	0.02		1.45 (1.02, 2.06)	0.04
≥10		1.47 (1.04, 2.08)	0.03		1.64 (1.17, 2.29)	<0.001
ADHD-I subtype (DSM-5)						
0		1 [Reference]			1 [Reference]	
1 to <5		1.31 (1.22, 1.41)	<0.001		1.24 (1.14, 1.34)	<0.001
5 to <10		1.40 (1.24, 1.58)	<0.001		1.44 (1.31, 1.58)	<0.001
≥10		1.36 (1.24, 1.50)	<0.001		1.40 (1.28, 1.54)	<0.001
ADHD-C subtype (DSM-5)						
0		1 [Reference]			1 [Reference]	
1 to <5		1.24 (1.07, 1.44)	<0.001		1.14 (0.97, 1.35)	0.12
5 to <10		1.61 (1.29, 2.01)	<0.001		1.49 (1.24, 1.78)	<0.001
≥10		1.53 (1.28, 1.82)	<0.001		1.64 (1.38, 1.95)	<0.001

Abbreviations: ADHD, attention-deficit/hyperactivity disorder; ADHD-C, ADHD with combined symptoms of inattention and hyperactivity-impulsivity; ADHD-HI, ADHD with symptoms of hyperactivity-impulsivity; ADHD-I, ADHD with symptoms of inattention; CI, confidence interval; DSM-5, the Diagnostic and Statistical Manual of Mental Disorders-Fifth Edition; OR, odd ratios; SHS, secondhand smoke.

[a] The definition of ADHD using the DSM-5 criteria could be defined using the Chinese version of the symptom inventory scale of ADHD. Children with ADHD symptoms were defined as presenting often or very often with 5 or more symptoms of inattention, or more symptoms of hyperactivity-impulsivity, or both. The ADHD symptom were further classified into 3 subtypes: ADHD-I, defined as presenting often or very often with 5 or more symptoms of inattention; ADHD-HI, defined as presenting often or very often with 5 or more symptoms of hyperactivity-impulsivity; and ADHD-C, defined as presenting often or very often with 5 or more symptoms of both inattention and 5 or more symptoms of hyperactivity-impulsivity

[b] The results were shown using the adjusted models with the adjustment of school-level random intercepts, child age, sex, only child, preterm birth and low birth weight, parental educational levels, yearly household income, maternal age, current and prenatal maternal smoking and prenatal maternal alcohol consumption.

© 2021 Lin LZ et al. *JAMA Network Open*.

eTable 6. The Associations of SHS Exposure From Pregnancy to Childhood With ADHD Symptoms and Subtypes in School-Aged Children (6-18 Years) Using the Continuous Scores of the Symptom Inventory Scale of ADHD and C-ASQ [a]

ADHD symptoms and subtypes	Adjusted model [b]	
	Estimates (95% CI)	*P* value
ADHD symptoms total score		
Unexposed	1 [Reference]	
Ever exposed	1.58 (1.44, 1.72)	<0.001
Always exposed	3.62 (3.39, 3.84)	<0.001
Inattention sub-scores		
Unexposed	1 [Reference]	
Ever exposed	0.98 (0.89, 1.07)	<0.001
Always exposed	2.32 (2.17, 2.46)	<0.001
hyperactive/impulsive sub-scores		
Unexposed	1 [Reference]	
Ever exposed	0.60 (0.54, 0.66)	<0.001
Always exposed	1.30 (1.20, 1.40)	<0.001
C-ASQ score		
Unexposed	1 [Reference]	
Ever exposed	0.78 (0.71, 0.86)	<0.001
Always exposed	1.76 (1.63, 1.88)	<0.001
C-ASQ Z-score		
Unexposed	1 [Reference]	
Ever exposed	0.08 (0.07, 0.09)	<0.001
Always exposed	0.18 (0.16, 0.19)	<0.001

Abbreviations: ADHD, attention-deficit/hyperactivity disorder; CI, confidence interval; OR, odd ratios; SHS, secondhand smoke.

[a] The results were shown using the adjusted models with the adjustment of school-level random intercepts, child age, sex, only child, preterm birth and low birth weight, parental educational levels, yearly household income, maternal age, current and prenatal maternal smoking and prenatal maternal alcohol consumption.

© 2021 Lin LZ et al. *JAMA Network Open*.

eTable 7. The Associations of SHS Exposure With ADHD Symptoms and Subtypes in Different Timing in School-Aged Children (6-18 Years) Using the Continuous Scores of the Symptom Inventory Scale of ADHD and C-ASQ [a]

ADHD symptoms and subtypes	Adjusted model [b]	
	Estimates (95%CI)	*P* value
Prenatal (reference: prenatal unexposed)		
ADHD symptoms total score	2.67 (2.50, 2.84)	<0.001
Inattention sub-scores	1.71 (1.61, 1.82)	<0.001
hyperactive/impulsive sub-scores	0.96 (0.88, 1.03)	<0.001
C-ASQ score	1.33 (1.24, 1.43)	<0.001
C-ASQ Z-score	0.13 (0.12, 0.14)	<0.001
Postnatal (reference, postnatal unexposed)		
ADHD symptoms total score	1.83 (1.62, 2.05)	<0.001
Inattention sub-scores	1.13 (1.00, 1.27)	<0.001
hyperactive/impulsive sub-scores	0.70 (0.60, 0.79)	<0.001
C-ASQ score	0.92 (0.80, 1.04)	<0.001
C-ASQ Z-score	0.09 (0.08, 0.10)	<0.001
Current (reference: current unexposed)		
ADHD symptoms total score	0.90 (0.76, 1.04)	<0.001
Inattention sub-scores	0.56 (0.47, 0.65)	<0.001
hyperactive/impulsive sub-scores	0.34 (0.28, 0.40)	<0.001
C-ASQ score	0.41 (0.33, 0.49)	<0.001
C-ASQ Z-score	0.04 (0.03, 0.05)	<0.001

Abbreviations: ADHD, attention-deficit/hyperactivity disorder; CI, confidence interval; OR, odd ratios; SHS, secondhand smoke.

[a] The results were shown using the adjusted models with the adjustment of school-level random intercepts, child age, sex, only child, preterm birth and low birth weight, parental educational levels, yearly household income, maternal age, current and prenatal maternal smoking and prenatal maternal alcohol consumption.

© 2021 Lin LZ et al. *JAMA Network Open*.

eTable 8. The Associations of Current Paternal Smoking and ADHD Symptoms and Subtypes in School-Aged Children (6-18 Years) Using the Continuous Scores of the Symptom Inventory Scale of ADHD and C-ASQ [a]

Numbers of cigarettes per day	Weekdays			Weekends	
	Estimates (95%CI)	*P* value		Estimates (95%CI)	*P* value
ADHD symptoms total score					
0	1 [Reference]			1 [Reference]	
1 to <5	1.30 (1.14, 1.47)	<0.001		1.15 (0.97, 1.32)	<0.001
5 to <10	1.47 (1.19, 1.74)	<0.001		1.48 (1.27, 1.69)	<0.001
≥10	1.31 (1.10, 1.52)	<0.001		1.47 (1.26, 1.69)	<0.001
Inattention sub-scores					
0	1 [Reference]			1 [Reference]	
1 to <5	0.84 (0.73, 0.94)	<0.001		0.73 (0.62, 0.85)	<0.001
5 to <10	0.90 (0.73, 1.08)	<0.001		0.95 (0.81, 1.08)	<0.001
≥10	0.84 (0.71, 0.98)	<0.001		0.94 (0.80, 1.07)	<0.001
hyperactive/impulsive sub-scores					
0	1 [Reference]			1 [Reference]	
1 to <5	0.47 (0.40, 0.54)	<0.001		0.41 (0.34, 0.49)	<0.001
5 to <10	0.56 (0.44, 0.68)	<0.001		0.53 (0.44, 0.62)	<0.001
≥10	0.46 (0.37, 0.55)	<0.001		0.54 (0.44, 0.63)	<0.001
C-ASQ score					
0	1 [Reference]			1 [Reference]	
1 to <5	0.61 (0.52, 0.70)	<0.001		0.53 (0.44, 0.63)	<0.001
5 to <10	0.74 (0.59, 0.89)	<0.001		0.71 (0.59, 0.82)	<0.001
≥10	0.62 (0.51, 0.74)	<0.001		0.72 (0.60, 0.84)	<0.001
C-ASQ Z-score					
0	1 [Reference]			1 [Reference]	
1 to <5	0.06 (0.05, 0.07)	<0.001		0.05 (0.04, 0.06)	<0.001
5 to <10	0.07 (0.06, 0.09)	<0.001		0.07 (0.06, 0.08)	<0.001
≥10	0.06 (0.05, 0.07)	<0.001		0.07 (0.06, 0.08)	<0.001

Abbreviations: ADHD, attention-deficit/hyperactivity disorder; CI, confidence interval; OR, odd ratios.

[a] The results were shown using the adjusted models with the adjustment of school-level random intercepts, child age, sex, only child, preterm birth and low birth weight, parental educational levels, yearly household income, maternal age, current and prenatal maternal smoking and prenatal maternal alcohol consumption.

© 2021 Lin LZ et al. *JAMA Network Open*.

eTable 9. The Associations of SHS Exposure in Different Timing With ADHD Symptom and Subtypes in School-Aged Children (6-18 Years) [a, b]

SHS exposure	OR (95%CI)			
	ADHD	ADHD-HI	ADHD-I	ADHD-C
Never exposed	1 [Reference]	1 [Reference]	1 [Reference]	1 [Reference]
Only current exposed	1.24 (1.10, 1.40)	1.49 (0.84, 2.63)	1.21 (1.06, 1.38)	1.39 (1.00, 1.94)
P value	<0.001	0.17	<0.001	0.05
Only postnatal exposed	1.71 (1.28, 2.29)	2.68 (0.81, 8.88)	1.59 (1.15, 2.20)	2.20 (1.06, 4.55)
P value	<0.001	0.11	<0.001	0.03
Only postnatal and current exposed	1.63 (1.32, 2.00)	3.24 (1.47, 7.14)	1.48 (1.17, 1.88)	2.19 (1.30, 3.70)
P value	<0.001	<0.001	<0.001	<0.001
Only prenatal exposed	2.00 (1.53, 2.63)	2.57 (0.78, 8.48)	2.09 (1.57, 2.79)	1.13 (0.41, 3.08)
P value	<0.001	0.12	<0.001	0.81
Only prenatal and current exposed	1.95 (1.54, 2.48)	1.93 (0.58, 6.34)	2.00 (1.55, 2.59)	1.65 (0.80, 3.40)
P value	<0.001	0.28	<0.001	0.18
Only prenatal and postnatal exposed	2.36 (1.93, 2.88)	4.34 (1.95, 9.66)	2.22 (1.79, 2.77)	2.75 (1.62, 4.66)
P value	<0.001	<0.001	<0.001	<0.001
Always exposed	2.89 (2.56, 3.26)	2.45 (1.30, 4.65)	2.90 (2.54, 3.30)	2.96 (2.11, 4.15)
P value	<0.001	0.01	<0.001	<0.001

Abbreviations: ADHD, attention-deficit/hyperactivity disorder; ADHD-C, ADHD with combined symptoms of inattention and hyperactivity-impulsivity; ADHD-HI, ADHD with symptoms of hyperactivity-impulsivity; ADHD-I, ADHD with symptoms of inattention; CI, confidence interval; OR, odd ratios; SHS, secondhand smoke.

[a] The Chinese version of the symptom inventory scale of ADHD consists of 18 queries categorized as 9 symptoms of inattention and 9 symptoms of hyperactivity-impulsivity. Each item or symptom was rated on a 4-point Likert scale (0 = never or rare; 1 = sometimes; 2 = often and 3 = very often). Children with ADHD

© 2021 Lin LZ et al. *JAMA Network Open*.

symptoms were defined as presenting often or very often with 6 or more symptoms of inattention, or more symptoms of hyperactivity-impulsivity, or both. The ADHD symptom were further classified into 3 subtypes: ADHD-I, defined as presenting often or very often with 6 or more symptoms of inattention; ADHD-HI, defined as presenting often or very often with 6 or more symptoms of hyperactivity-impulsivity; and ADHD-C, defined as presenting often or very often with 6 or more symptoms of both inattention and 6 or more symptoms of hyperactivity-impulsivity.

[b] The results were shown using the adjusted models with the adjustment of school-level random intercepts, child age, sex, only child, preterm birth and low birth weight, parental educational levels, yearly household income, maternal age, current and prenatal maternal smoking and prenatal maternal alcohol consumption.

© 2021 Lin LZ et al. *JAMA Network Open*.
